# Recent progress in the fabrication of nanocomposite-derived superhydrophobic coatings for improved concrete lifespan: insights into recent advancements, interfacial chemistry and tribological performance

**DOI:** 10.1039/d5ra09515h

**Published:** 2026-03-10

**Authors:** Sahita Karmakar, Pranav R. T. Peddinti, Young-Nam Kwon, Saikat Sinha Ray

**Affiliations:** a Department of Environmental Science and Engineering, SRM University-AP Amaravati Andhra Pradesh 522240 India ssinharay6@gmail.com; b Department of Civil Engineering, SRM University-AP Amaravati Andhra Pradesh 522240 India; c Department of Civil, Urban, Earth, and Environmental Engineering, Ulsan National Institute of Science and Technology (UNIST) Ulsan 44919 Republic of Korea

## Abstract

Concrete structures and built infrastructure are essential for modern civilization due to the increasing urbanization, population growth, and corresponding infrastructure needs. However, extreme weather and prolonged exposure to humidity can lead to the irreversible degradation of various materials used in built infrastructure, primarily due to water penetration and freeze–thaw cycles. This review examines the effectiveness and successful application of protective surface coatings on concrete, a major construction material. Superhydrophobic coatings meet the demands of various applications, offering anti-microbial resistance, corrosion resistance, contamination prevention, water repellence, drag reduction, anti-icing, anti-fogging, anti-adhesion features, and self-cleaning capabilities. Nanocomposites are known for their improved mechanical, thermomechanical, and thermal properties compared with their base materials, drawing considerable interest from researchers worldwide. The key problem of insufficient mechanical and chemical durability of superhydrophobic materials when applied on concrete surfaces is pointed out, and the application trend of these materials is projected. In addition, the tribology discussed in this review primarily addresses their mechanical durability, wear resistance, and long-term performance. This review also covers recent advancements in fabrication techniques, current limitations, and testing methods for superhydrophobic concrete. The effects of hydrophobic treatments on the hydration process, which is crucial for the development of compressive strength in concrete, are explored. Furthermore, successful case studies of superhydrophobic coatings for protecting concrete structures are presented. To develop maintenance strategies, prevent failures, and adhere to industrial standards, it is vital to assess the durability and characteristics of these coatings while considering factors such as mechanical stress, environmental exposure, and other qualitative aspects. The overall findings from this review could facilitate enhancing the advancement of durable, efficient concrete materials.

## Abstract 2

Superhydrophobic coatings show significant potential for concrete structures and heritage protection because of their excellent wetting resistance.

## Graphical abstract

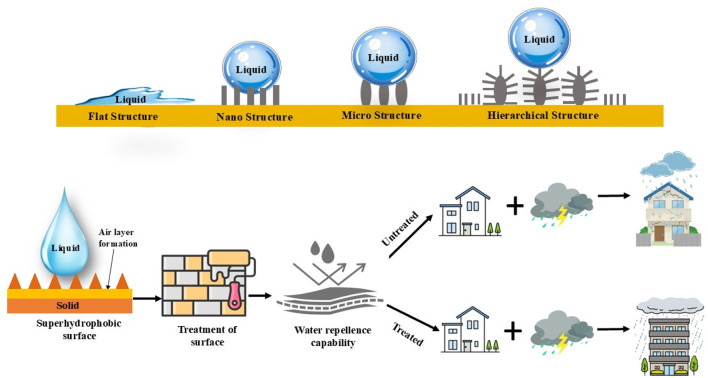

## 1 Introduction

Concrete structures and building materials intrinsically impact the daily lives and safety of residents, significantly influencing urban growth and the reputation of construction enterprises. Urban infrastructures suffer from considerable damages due to pollution from dust, organic pollutants in the air, and microbes, which degrade concrete structures and create aesthetic deterioration.^1^ Water seepage in concrete structures has caused significant challenges and disruptions for societies globally. Waterproofing issues are recognized as the primary cause of structural failures, leading to moisture problems.^2^ Recently, the coating industry has gained significant attention. Super-hydrophobic coatings are employed in the building sector because of their remarkable qualities, offering various functional benefits such as anti-microbial resistance, anti-fogging, anti-corrosion, anti-fouling, self-cleaning, and drag reduction capabilities. Furthermore, customizing the properties of these coatings to meet important performance standards, such as transparency, UV resistance, anti-reflection, anti-water penetration, and thermal insulation, using various fabrication technologies and materials has enhanced their applications across diverse fields.^3^

**Fig. 1:**
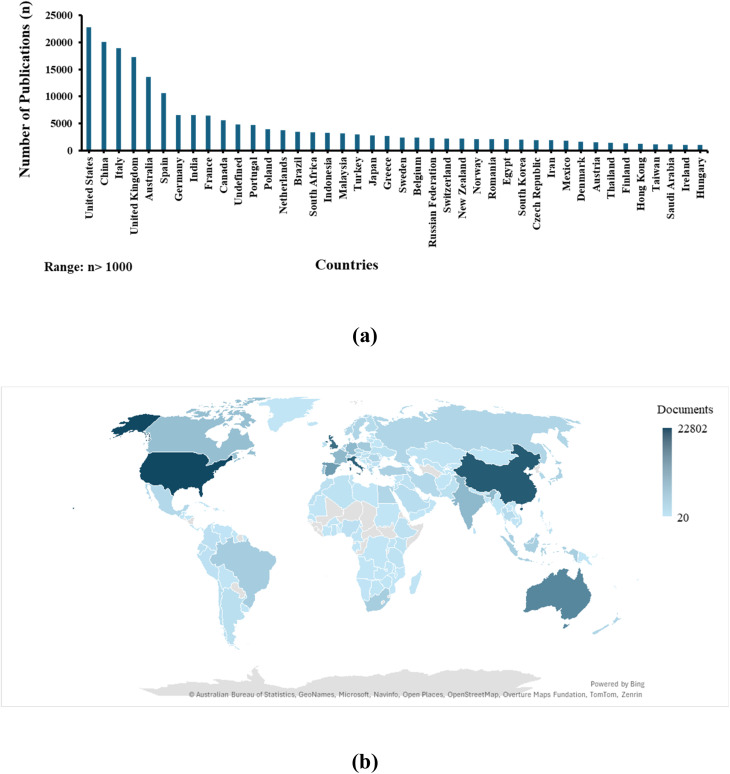
Global geographical distribution of scientific publications in conserving their heritage. The database was collected from an advanced search in Scopus using the terms “conservation” and “heritage” from 2005 to 2025 as of February 2026.

Fig. 1 represents the country-wise research productivity related to the conservation of heritage structures, as identified from the literature. Fig. 1(a) shows the distribution of global research outputs from different countries that have more than a thousand publications related to conservation of heritage sites. It visualizes the top countries involved in research and development worldwide in conserving their heritage based on a comprehensive dataset collected from an advanced search in Scopus. The United States, China, Italy, and United Kingdom stand out as global leaders, exhibiting the highest levels of heritage conservation activity.

**Fig. 2:**
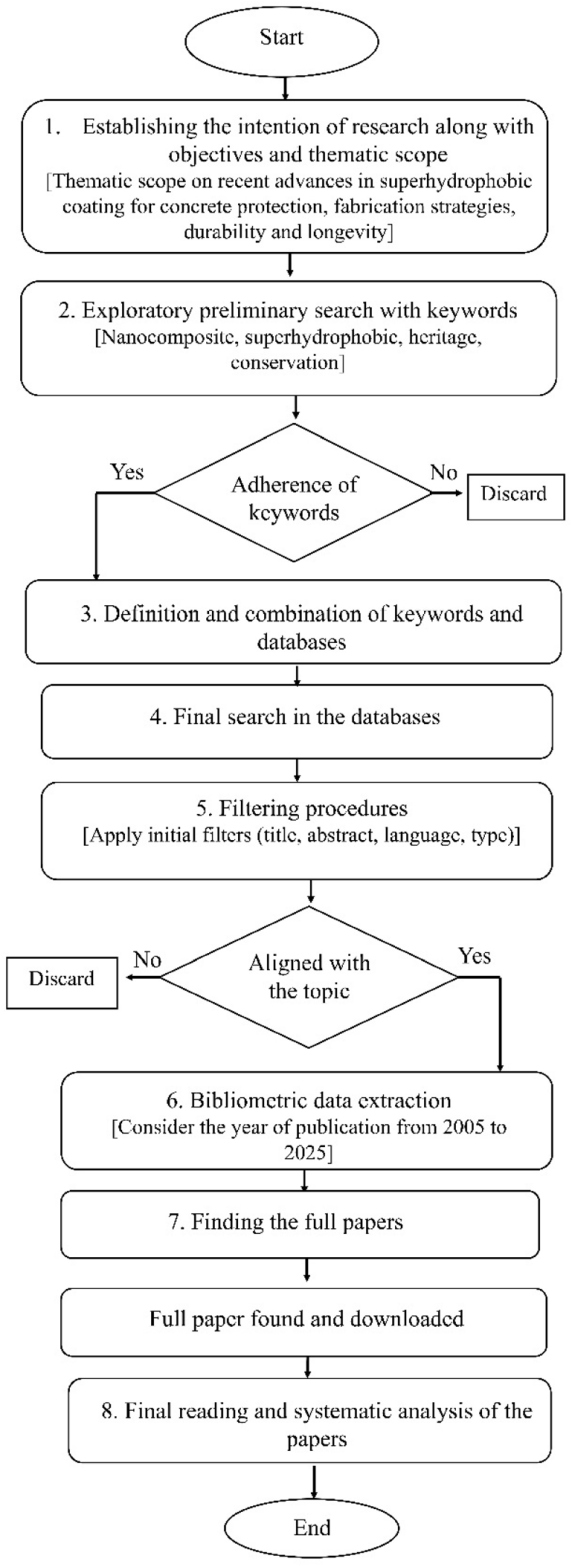
Bibliometric analysis of the methodology for the literature search strategy.

The methodology for conducting the comprehensive literature review on nanocomposite-based superhydrophobic coatings is shown in Fig. 2 using bibliometric analysis.^4,5^ Step 1 defines the research intention and objectives. In step 2, a set of relevant keywords is identified. In step 3, the identified keywords are used in different combinations that can help to find the required literature, for example, ‘Nanocomposite’ OR ‘Superhydrophobic’ AND ‘Heritage’ OR ‘Conservation’. Then, the literature search is conducted, and the dataset is downloaded. This phase collects peer-reviewed publications from established databases, such as Scopus, Web of Science, ScienceDirect, and ResearchGate, to build a custom dataset that captures a broad range of relevant publications. Regarding the databases, only those that provide a larger number of works on the research topic were selected. Step 4 usually includes a final database search using Zotero Reference Manager. In step 5, the articles are analysed individually, excluding titles unrelated to the area. Typically, a temporal limit is imposed to customise the literature review in step 6. Determining whether the full paper is available for download is the next step. The final phase includes reading and systematic analysis of the papers collected thereafter. This can be one of the fundamental approaches of bibliometric analysis in this research area.

Typically, there are two main approaches for achieving superhydrophobicity in concrete, namely, surface coating and integrated modification; however, they face distinct challenges in maintaining a balance between the robustness and mechanical strength of the resulting concrete.^6^ Superhydrophobic coatings, which rely on low surface energy micro- and nano-scale structures, are particularly vulnerable to structural degradation under localized load accumulation, leading to the collapse of trapped air cushions at the solid–liquid interface.^7^ When this protective surface structure is compromised by mechanical wear, chemical corrosion, or environmental aging, it exposes the underlying hydrophilic concrete substrate. Superhydrophobic coatings show significant potential for heritage protection because of their excellent wetting resistance. As emerging technology, these coatings can help safeguard artifacts from damage caused by acid rain and precipitation. Their low surface energy and roughness further enhance their wetting resistance. Chemical materials such as silicones and fluoropolymers are commonly used to achieve low surface energy.^8^

**Fig. 3:**
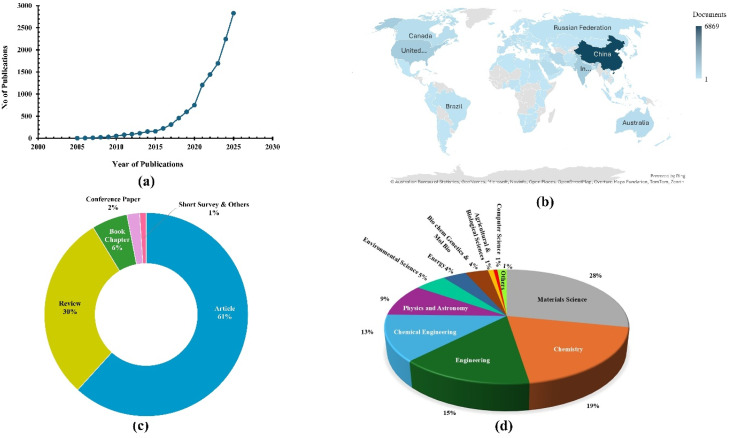
(a) Survey on the number of research papers published year-wise since 2005. (b) Number of documents published from different countries. (c) Different categories of publications. (d) Contributions from different subject areas. These databases were collected from an advanced search in Scopus with the terms “superhydrophobic” and “nanocomposite for building” as of February 2026.

Fig. 3 illustrates the country-wise distribution of research productivity related to superhydrophobic nanocoating materials for building protection. A survey has been conducted on peer reviewed publications since 2005 from different countries, demonstrating the growing interest in research articles on the superhydrophobic modification of nano-based materials. As shown in Fig. 3, China leads significantly with the highest number of publications, highlighting its strong research focus in this domain. Other countries, such as India, the United States, Iran, South Korea, and the United Kingdom, also exhibit moderate research activity in building conservation and related construction-material applications. This emerging interdisciplinary field addresses the growing need for innovative materials to combat structural degradation due to environmental and chemical exposure. Numerous researchers have systematically explored superhydrophobic nano-based materials from various angles, including surface or matrix hydrophobic modification, performance evaluation, mechanism analysis, development of hydrophobic agents, and engineering applications.

Composite and nanocomposite materials are made from different matrices, such as polymers, carbon, metals, and ceramics, combined with reinforcements such as particles, fibers, and layered materials.^9^ Compared to traditional polymer composites, polymer nanocomposites exhibit enhanced mechanical and tensile strength, superior scratch resistance, a higher heat distortion temperature, and effective noise reduction.^10^ Common challenges associated with high reinforcement levels in composites, such as lower toughness, decreased optical clarity, and increased melt viscosity, are minimized in nanocomposite production, as a nano-reinforcement loading of less than 10 wt% is sufficient for creating high-performance polymer nanocomposites.^11^ The longevity of nanocomposite-based protective coatings is crucial for decreasing maintenance expenses and maximizing the service life of structures.

These coatings are subjected to mechanical stresses, such as abrasion and impact, which exacerbate wear. Therefore, reliable inspection techniques are required to monitor the performance of coatings throughout their lifetimes.^12^ Historically, evaluations of coatings have predominantly relied on visual inspections and invasive testing techniques, including cross-cut adhesion assessments and pull-off strength measurements (Ovie *et al.*, 2024).^13^ These techniques provide direct and quantifiable results; they often require localized sampling, which can compromise structural integrity.^14^

**Fig. 4:**
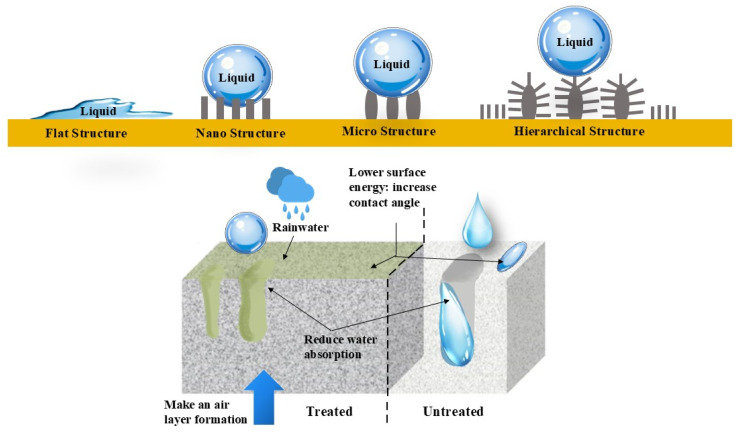
Self-cleaning behavior of surfaces treated with and without nanocomposite-based superhydrophobic coating materials.

Currently, research focuses on the superhydrophobic modification of nano-based materials aimed at preserving concrete structures and heritage sites. However, there is a significant lack of standardized and affordable superhydrophobic coatings that guarantee durability.^15^Fig. 4 illustrates the properties and effects of a surface treatment by comparing treated and untreated surfaces. The untreated surface is smooth, allowing liquids (*e.g.*, rainwater) to spread, resulting in lower contact angles and higher surface energy. In contrast, the treated surface exhibits a lower surface energy and higher contact angles, which enable it to repel water. Additionally, this surface features nano- or micro-scale roughness, which traps air and decreases the interaction between the liquid and solid.^16,17^ These coatings are designed to provide excellent water repellency, self-cleaning properties, and resistance to environmental degradation by utilizing affordable nanomaterials and scalable production techniques, making them suitable for a broader range of applications.^18^ This approach not only extends the lifespan of valuable architectural and cultural assets but also promotes environmentally conscious preservation by reducing maintenance needs and reliance on harmful chemicals.^19^

This review concentrates on the effectiveness and synthesis of superhydrophobic nanocomposite-based coating materials for preserving concrete structures while discussing the classification and synthesis processes of these coatings. Their fabrication techniques and durability tests are enhanced to assess their water repellency capabilities. It also discusses the tribology performance and durability of nano-composite-based superhydrophobic coatings under mechanical stress due to their micro/nano-structured surfaces, which enable extremely low wettability and improved resistance to friction, wear, and deformation. These coatings maintain their water-repellent properties, reduce friction coefficients, and minimize material loss even under repeated abrasion, making them ideally suited for long-term applications.

Furthermore, this review addresses sustainability challenges, focusing on eco-friendly materials and scalable production methods, and provides techno-economic analyses to assess the cost-effectiveness of these approaches for large-scale applications. Despite the significant advancements in the fabrication of nanocomposite-derived superhydrophobic coatings for enhancing the lifespan of concrete, several research gaps persist, particularly in areas of long-term durability, real-world applicability, and interfacial chemistry. One major gap is the limited understanding of their long-term mechanical and chemical stability under harsh environmental conditions. Additionally, gaps remain in field testing and scalability for practical applications, with many formulations showing promise in laboratory settings but lacking data on real-world performance. Ultimately, a research gap remains regarding the techno-economic factors of nanomaterial-based techniques, which tend to be expensive. Therefore, future research must emphasize improving both durability and cost-effectiveness.

## 2 Fundamentals of superhydrophobicity for concrete coatings

Inspired by the “lotus effect,” superhydrophobic surfaces effectively repel water. Specifically, low-adhesion superhydrophobic surfaces with a high-water contact angle and reduced contact angle hysteresis enhance the self-removal of liquid and particulate contaminants.^20,21^ Their unique properties make them ideal for applications including anti-fogging, self-cleaning, anti-icing, heat transfer enhancement, and energy harvesting. Recent developments in coating technologies, especially the advent of smart superhydrophobic coatings, have made these materials multifunctional and highly efficient, attracting considerable interest among researchers globally.^7^ Furthermore, these innovations have expanded the potential applications of superhydrophobic coatings by improving essential performance traits through various fabrication techniques and materials. Key characteristics include transparency, UV resistance, anti-reflective features, water penetration resistance, thermal insulation, and flame retardancy.^22^

### 2.1 Surface properties governing superhydrophobicity

**Fig. 5:**
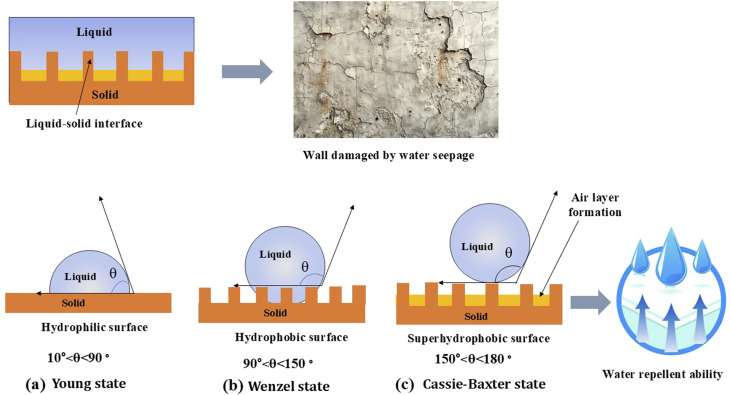
Diagram illustrating various surface wettability through contact angle measurements.

A superhydrophobic surface has a high-water contact angle (WCA) exceeding 150° and a low contact angle hysteresis (CAH) or sliding angle (SA) below 10°. Fig. 5 depicts that the wetting characteristics of these surfaces can be described using the theoretical models proposed by Young, Wenzel, and Cassie–Baxter.^23^ The Young model represents thermodynamic equilibrium among solid, liquid, and vapor phases on an ideal, smooth, and uniform solid surface. In the Cassie–Baxter model, for example, a droplet rests on the tips of surface protrusions, with air pockets trapped beneath. In contrast, the classical Wenzel model depicts liquid droplets contacting every point on the underlying solid surface.^24^ The wettability of materials is determined by their surface-free energy and surface roughness, which can be explained through the principle of surface hydrophobicity. Wettability refers to the ease with which a surface absorbs or repels water.^25^ On surfaces with low wettability, water cannot infiltrate, which can prevent structural damage, staining, or the growth of mold and algae. High contact angle surfaces (greater than 150°, typical for superhydrophobic surfaces) demonstrate excellent water repellency, preventing moisture ingress, freeze–thaw cycles, and the accumulation of pollutants.^22^ Surface roughness is crucial in preserving construction and heritage materials, directly affecting their interaction with water and contaminants. The roughness is significantly enhanced by creating micro- or nano-scale textures, which improve the hydrophobic or superhydrophobic properties, allowing water beads to roll off rather than penetrate the surface.^26^

### 2.2 Technological and engineering applications of superhydrophobic coatings

**Fig. 6:**
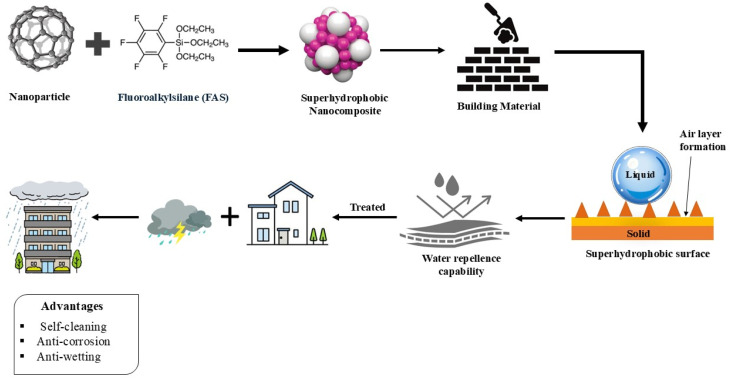
Advantages of using superhydrophobic nanocomposites for coating the surfaces of concrete structures.

Superhydrophobic surfaces achieve their protective properties through tailored surface chemistry, hierarchical micro/nanoscale roughness, and advanced material engineering. These mechanisms work synergistically to repel water, resist corrosion, enable self-cleaning, and withstand UV degradation.^27^ The extent to which a solid can repel a liquid relies on two key factors, its surface energy and surface morphology. A reduction in surface energy enhances its hydrophobicity.The chemical composition of a surface determines its surface energy, which in turn strongly affects its wettability. In a superhydrophobic surface, its surface morphology plays a crucial role in determining its wettability.^28^ Roughening a surface can intensify its hydrophobicity not only because of the increased solid–liquid interface but also because air can be trapped between the rough surface and a liquid droplet. Since the air layer acts as a hydrophobic material with a contact angle 180°, air trapping boosts the surface hydrophobicity. Thus, the hierarchical micro- and nano-structuring of a surface is responsible for superhydrophobicity. Hydrophobic surface materials, introduced by the fluoropolymers, combine with nanoscale roughness to produce high water contact angles, repelling moisture.^29^Fig. 6 illustrates the fabrication and advantages of superhydrophobic surfaces made up of fluoroalkylsilane (FAS)-incorporated nanocomposites. Such materials are used in self-cleaning coatings, anti-corrosion layers, and water-resistant surfaces. Table 1 provides a detailed overview of various coating materials used for hydrophobic applications, along with their protection mechanisms, key chemistry involved, and applications. The materials used for hydrophobic coatings, include nano-silica (SiO_2_), nano-titanium dioxide (TiO_2_), and polymers such as PMMA (polymethyl methacrylate) and PDMS (polydimethylsiloxane). Moreover, Table 1 describes how each material achieves hydrophobicity, such as through surface roughness, chemical modification, or forming protective layers.

**Table 1:** Overview of the superhydrophobic coating materials and their applications on concrete surfaces

Coating material	Mechanism of protection	Key chemistry involved	Application	Reference
Nano-silica (SiO_2_) incorporated with PDMS	Surface roughness at the nanoscale and low surface energy due to chemical modifications	These agents react with the silanol groups on the nano-silica surface, replacing them with hydrophobic groups	Improves UV aging resistance, saltwater immersion performance	3
Nano-titanium dioxide (TiO_2_) modified by fluorinated compounds	Water repellence, dirt resistance, chemical and environmental protection	Treated with silane-based compounds, fluorinated alkyl chains, or other hydrophobic agents	Lowers surface energy, provides micro–nano roughness, enables high water contact angles (>150°), reducing water ingress, capillary absorption	30
Nano-calcium carbonate (CaCO_3_) combined with fluoropolymers	It provides a high surface area for interaction and dispersion in coatings or composites	These molecules attach to the surface of CaCO_3_*via* chemical or physical bonds, replacing hydrophilic groups with hydrophobic ones	Improved durability, self-cleaning properties	31
Hexamethyldisilazane (HMDS)	It can form a monolayer on surfaces, providing uniform coverage and consistent hydrophobicity	The trimethylsilyl groups create a low-energy surface, which minimizes the interaction between water molecules and the surface	Restricting water penetration, surface treatment to reduce water absorption	32
Polymethyl methacrylate (PMMA)	The presence of nonpolar methyl groups (–CH_3_) and ester group (–COOCH_3_) in its polymer backbone. These groups lack polar or hydrophilic functional moieties, resulting in low affinity for water molecules	It is composed of long chains of methyl methacrylate monomers, which create a highly stable structure	Good tensile strength, protective coating, binder, and surface modifier	33
Trimethylchlorosilane (TMCS)	Mechanism based on its chemical reactivity with hydroxyl groups (–OH) present on surfaces, such as silica, glass, or cellulose, forming a hydrophobic silane layer	TMCS reacts *via* a silylation process, where its silicon atom forms a covalent bond with the oxygen of the hydroxyl group, releasing hydrochloric acid (HCl) as a byproduct	Used as a silylating agent to modify surfaces by attaching hydrophobic trimethylsilyl (TMS) groups and porous materials. It is employed for surface hydrophobization and protective treatments	34
Tetraethylorthosilicate (TEOS) mixed with fluoroalkyl silane (FAS)	The hydrophobic layer formed by the functionalized silica network resists moisture penetration and prevents water interaction with the substrate	The silanol groups formed during hydrolysis undergo condensation, leading to the formation of Si–O–Si bonds (siloxane bonds) and the release of water or ethanol	Used in protective coatings, antifouling surfaces, microelectronics and sensors, and automotive and aerospace applications	35
Polydimethylsiloxane (PDMS)	When applied to a substrate, PDMS forms a thin, uniform film that acts as a barrier to water and moisture	The –CH_3_ groups on the PDMS polymer are nonpolar and repel water molecules, minimizing water interaction with the surface	Used in spin-coating, dip-coating, or spray coating, followed by curing to form a stable hydrophobic layer	25

### 2.3 Anti-microbial superhydrophobic coatings for concrete structures

Microbial activity can influence the durability of building materials through various mechanisms of biodeterioration.^36^ The tendency of microbial activity depends on the type and extent of microbial growth, the type of colonised material, and the level of pollution.^37^ Antimicrobial superhydrophobic coatings on concrete structures provide dual benefits by both repelling water and resisting microbial growth.^38^ It has been observed that their decline in structural capacity over time primarily results from chloride ingress *via* six transport processes: adsorption, diffusion, binding, penetration, capillary action, and dispersion. This process causes steel corrosion, concrete cracking, loss of bond and spalling.^6^ Among the causes of structural decay, biologically induced deterioration is notably significant in harsh environments, a phenomenon known as microbiologically induced concrete corrosion (MICC).^39^ This is a major concrete-damaging process that significantly threatens the durability and lifespan of sanitary sewer infrastructure elements, including pipes, junctions, manholes, and pump stations, leading to substantial cost implications.^40^ The main groups associated with MICC include sulphate-reducing bacteria and archaea, acid-producing bacteria, thiosulphate-reducing bacteria, iron-reducing bacteria, iron-oxidising bacteria, nitrate-reducing bacteria, and methanogenic archaea.^41^

It has been reported that antimicrobial agents work through various mechanisms, such as electrostatic interactions, the production of reactive oxygen species (ROS), and the release of metal ions or metal nanoparticle ions.^42,43^ These mechanisms typically target and weaken the bacterial cell wall, leading to bacterial cell death.^44^

**Fig. 7:**
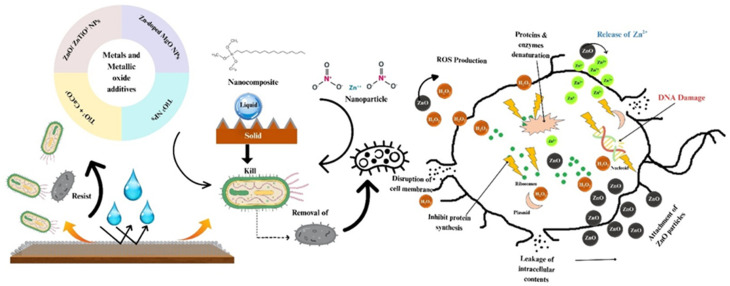
Schematic of the antimicrobial mechanism of a superhydrophobic surface based on ZnO nanoparticles.

ZnO nanoparticles have demonstrated antimicrobial activity against various microbial species.^45^ Kumar *et al.* reported that ZnO can chemically and physically interfere with bacteria and their further growth. Chemically, ZnO has the ability to react with the bacterial cell, thereby producing photo-induced ROS, and form and release H_2_O_2_ and Zn^2+^ ions, as shown in Fig. 7. These products damage the bacterial cell membrane and DNA, causing cell death.^46^ ZnO nanoparticle coatings have been found to improve the durability of stone surfaces against fungal attack (such as *Aspergillus niger* and *Penicillium* sp.) by generating ROS when exposed to UV light.^47^ Ruffolo and La Russa investigated the efficiency of ZnO and ZnTiO_3_ nanocoatings on stone heritage samples and found that ZnTiO_3_ provided higher inhibition than ZnO, even though both are hydrophobic and photocatalytic. The use of Zn-doped MgO nanoparticles (NPs) produced *via* the sol–gel method as antifungal coatings on dolomitic and calcite stones has been reported. These coatings aim to protect surfaces against fungi such as *Aspergillus niger*, *Penicillium oxalicum*, *Paraconiothyrium* sp., and *Pestalotiopsis maculans*, which are active in the bio-weathering of stone.^48^

In another instance, Yang *et al.* described a superhydrophobic coating made from fluorinated silica nanocomposites, which include fumed nano-SiO_2_ and fly-ash drifting beads (FDB). They are modified with 1*H*,1*H*,2*H*,2*H*-perfluorooctyltrimethoxysilane (FAS-13) and used with poly(vinyl alcohol) as a binder. The coating is applied using a low-pressure sprayer to penetrate concrete pores through capillary action. The optimized coating achieves a water contact angle of 162.4° and sliding angle of 3°, reducing water absorption by 72.03% in deionized water and 80.11% in 3.5 wt% NaCl solution over seven days. Antibacterial evaluations using the spread plate method reveal the reduced adhesion of *Escherichia coli* and *Staphylococcus epidermidis*, with inhibition rates of 84.57% and 90.43% respectively, which is attributed to the non-wetting surface preventing biofilm formation. This coating offers a practical, eco-friendly strategy for enhancing concrete durability in challenging environments.^49^

Titanium dioxide (TiO_2_) is recognised as a photocatalyst and is used in self-cleaning tiles, glass, windows, *etc.*^50^ Vishwakarma *et al.* reported that fly ash modified with TiO_2_ nanoparticles as well as mortar with TiO_2_ + CaCO_3_ NPs, exhibited its efficacy in antibacterial activities against *Pseudomonas*, *Fusarium*, algae, blue-green algae, and manganese oxidizing bacteria. Vishwakarma *et al.* concluded that adding 2% TiO_2_ to fly ash mortar enhanced antibacterial activity by reducing pH levels, and the oxidation of sulfur further helped inhibit bacterial growth.^51^

### 2.4 Concrete substrate compatibility and surface preparation

Superhydrophobic modification of concrete includes two approaches: surface superhydrophobic modification and bulk superhydrophobic modification.^52^ Surface superhydrophobic modification is typically achieved through coating or impregnation. Nevertheless, superhydrophobic coatings often suffer from drawbacks such as poor aging resistance, susceptibility to peeling, and inadequate breathability, which increase the costs associated with subsequent repairs.^53^ Bulk superhydrophobic modification involves directly incorporating low-surface-energy materials (such as silanes, siloxanes, and stearic acid) into the concrete mix to achieve an overall superhydrophobic effect in the concrete.^54^

For foamed concrete, most of the superhydrophobic research has been focused on surface modification, while less was on bulk modification due to the high difficulty of its preparation.^55^ Shi *et al.* utilized an isobutyltriethoxysilane (IBTS) and graphene oxide/silane (GO/IBTS) composite emulsion to perform coating and immersion treatments on foamed concrete, reducing its water absorption by 75% and 82%, respectively.^56^ Lu *et al.* developed a self-healing superhydrophobic coating using hydrophobic silica, silicone sealant, and epoxy resin. Gao *et al.* applied a silane emulsion to the surface of foamed concrete, endowing the specimens with excellent anti-icing properties.^57^

Table 2 presents a comparative analysis of the performance of various superhydrophobic coating systems applied to concrete, evaluating five different materials. This comparison highlights the trade-offs among the coatings, with some exhibiting excellent superhydrophobicity and others emphasising barrier protection or mechanical durability, thereby aiding the selection of appropriate systems to enhance the longevity of concrete against environmental degradation.

**Table 2:** Comparative performance of superhydrophobic coating systems for concrete

Coating material	Hydrophobicity/wettability	Barrier performance	Durability properties	References
Silane/siloxane	WCA 130°–145°	Water absorption reduction: 50–70%	Freeze thaw: good	58
	SA < 10°	Chloride ingress: 70–80%	Abrasion: poor	
	CAH low	Carbonation: moderate reduction	Adhesion: N/A	
Polyurethane-based coatings	WCA 90°–130°	Water absorption reduction: >90%	Freeze thaw: excellent	59
	SA < 10°	Chloride ingress: >85%	Abrasion: excellent	
	CAH medium–high	Carbonation: excellent reduction	Adhesion: 2–3 MPa (megapascals)	
Epoxy-based coatings	WCA 90°–140°	Water absorption reduction: >95%	Freeze thaw: excellent	60
	SA 5°–6°	Chloride ingress: >85%	Abrasion: excellent	
	CAH very high	Carbonation: excellent reduction	Adhesion: 2.5–3.5 MPa	
Fluorosilane-functionalized Al_2_O_3_	WCA 130°–160°	Water absorption reduction: >90%	Freeze thaw: excellent	61
	SA 7°–8°	Chloride ingress: >80%	Abrasion: good	
	CAH high	Carbonation: high reduction	Adhesion: 1.5–2 MPa	
PDMS-based nanocomposite coating	WCA 150°–158°	Water absorption reduction: 65–85%	Freeze thaw: good	62
	SA 6°–7°	Chloride ingress: >70%	Abrasion: good	
	CAH medium	Carbonation: moderate	Adhesion: 1–1.8 MPa	

Table 3 presents a mechanism-based comparison of the three major categories of concrete protection strategies commonly used to enhance durability and resist environmental degradation. It is an overview of a framework for understanding the trade-offs among different protection strategies and supports the informed selection of concrete protection systems based on exposure conditions and durability requirements.

**Table 3:** Mechanism-based comparison of major concrete protection strategies

Protection strategies	Mechanism of action	Typical penetration depth	Permeability/breathability	References
Film-forming surface coatings	Formation of a continuous barrier layer on the concrete surface; water repellency *via* surface roughness and low surface energy	50–500 µm (film thickness). Minimal substrate penetration	Often reduced vapor permeability; limited breathability	63
Penetrating hydrophobic impregnation/sealers	Diffusion of low-viscosity hydrophobic agents into pore walls, forming a water-repellent monolayer without pore blockage	1–10 mm (depth varies with substrate porosity, product viscosity, and application)	Largely preserved vapor permeability; good breathability	64
Pore-blocking/densification	Deposition of solid particles or precipitation of reaction products (*e.g.*, C–S–H) within the pore structure, physically reducing porosity and permeability	1–30 mm (can achieve significant depth for slurry-based or reactive penetrants)	Significantly reduced vapor permeability	60

## 3 Nanocomposite frameworks for designing superhydrophobic coatings

Nanocomposites are composite materials having one of their phases with dimensions in the nanometer range.^65^ Typically, they consist of multiple phases, with one phase within the nanoscale range of approximately 1 nm to 100 nm. At this scale, materials such as carbon, silicon, and metals display distinct physical properties, since 1 nm (1 × 10^−9^ m) is one-millionth of a millimeter.^66^ These nanocomposite materials are highly promising for creating superhydrophobic coatings with enhanced durability, reduced environmental impact, and improved self-cleaning capabilities.^67^ They are widely used in the packaging, aerospace, automotive, electronics, semiconductor, energy, construction, and cosmetics sectors. The incorporation of nanoparticles such as silica, titanium dioxide, or carbon nanotubes into a polymer matrix results in a rough surface with a chemical composition that imparts a super-low surface energy and high hydrophobicity, facilitating self-cleaning properties.^68^ Functionalization of these nanocomposites can lead to the development of water-repellent fabrics, anti-fogging surfaces, and corrosion-resistant coatings, showcasing their potential to enhance durability and functionality.^69^

### 3.1 Structural and functional classification of nanocomposites

Nanoparticles and nanofibers can be categorized according to their size, composition, aspect ratio, and electron microscopy images, which helps to elucidate their effects on nanocomposite properties.^70^ Due to these characteristics, nanocomposites are excellent substitutes for micro-composites and monolithic materials. They are made up of two or more distinct phases with different chemical and physical characteristics, separated by a clear interface.^71^ These composite materials have superior properties compared to conventional composite materials and have diverse applications in various areas, including surface coatings for the preservation of concrete and heritage structures.^72^ Nanocomposites are categorized based on the types of matrix materials used in their formulation. Typically, they fall into four main categories according to their matrix material: metal-based, polymer-based, carbon-based, and hybrid materials.^73^Table 4 focuses on the different types of nanocomposites used for superhydrophobic coatings, including their characteristics, advantages, typical reinforcements and case studies.

**Table 4:** Comparison of various case studies based on different types of nanocomposites

Types of nanocomposites	Matrix materials	Characteristics	Matrix reinforcements	Findings from case studies	Applications	References
Polymer-based nanocomposites (PMCs)	Polymers (*e.g.*, various resins, silanes)	Lightweight, corrosion resistance, improved thermal & mechanical properties	Nanoclay, carbon nanotubes (CNTs), graphene, nanosilica, metal oxides	Silane/clay nanocomposites (Cloisite 20A & I.30P); ∼50% reduction in moisture permeability; superior barrier with high-aspect-ratio Cloisite 20A	Self-cleaning, antimicrobial, antifouling coatings; concrete protection and reduced moisture permeability	74–79
Metal-based nanocomposites (MMNCs)	Metals or alloys (ductile matrix)	Enhanced mechanical strength, thermal stability, resistance to dislocation movement	Zero-dimensional (core–shell), one-dimensional (nanotubes), two-dimensional (laminar); various nanoparticles	Different reinforcement phases classified by *in situ vs. ex situ* fabrication methods for better uniformity/bonding	Tailored mechanical, catalytic, electrochemical properties *via in situ* and *ex situ* fabrication	75 and 80–83
Carbon-based nanocomposites (CNCs)	Carbon-based structures (*e.g.*, graphene, CNTs)	High surface area, tunable electrical & mechanical properties, functionalization improves dispersion	Graphene, CNTs, nanodiamonds, graphene oxide (GO), integrated with metal oxides/polymers	Functionalized graphene oxide (GO) forms hydrophobic protective coat; 83.4% corrosion inhibition efficiency at 20 mg L^−1^; graphene nanoplates (GNPs) create carbon-doped protective layer	Corrosion protection, long-term resistance in chloride-rich environments	84–88
Hybrid-material-based nanocomposites	Combination of organic & inorganic (polymeric, ceramic, metallic matrices)	Synergistic mechanical strength, chemical stability, UV resistance	CNTs, graphene, metal oxides (TiO_2_, ZnO, SiO_2_), metallic nanoparticles (Ag, Cu)	SiO_2_–TiO_2_ hybrid nanoparticles *via* sol–gel/alkoxide mixing; oxalic acid & surfactants prevent agglomeration/cracks; improved consolidation on carbonate stones	Self-cleaning, antimicrobial, UV-resistant coatings; crack-free protective coatings for stone surfaces	89–92

#### 3.1.1 Polymer-based nanocomposites

Polymer matrix nanocomposites (PMCs) are recognized as materials with notable characteristics. Polymers provide numerous benefits, including high durability, lightweight properties, ease of processing, resistance to corrosion, ductility, and affordability.^93^ However, they generally show inferior mechanical, thermal, and electrical properties compared to ceramics and metals. The physicochemical stability of nanocomposites (PMCs) presents a considerable challenge, mainly due to the complexity of the interfacial zones between the nanoparticles and the polymer matrices.^74^ These small scales create a large specific surface area, enhancing the significance of polymer–nanoparticle interactions. Due to their polymer-based matrix combined with nano-additives serving as reinforcing materials, these are known as polymer nanocomposites.^75,76^ The additives can be one-dimensional (such as nanotubes and fibers), two-dimensional (layered materials such as clay), or three-dimensional (in the form of spherical particles). The polymer matrix, whether incorporated or formed *in situ* with inorganic nanoparticles, has been extensively studied for developing nanostructures and enhancing waterborne coatings.^94^ The addition of inorganic nanoparticles can improve the waterproofing, mechanical, thermal, electrical, optical, or adhesive properties of the polymeric matrix, as well as some other functional properties.^95^ Martinez *et al.* developed photocatalytic coatings for building materials using TiO_2_ nanoparticles incorporated in a polymer matrix-based coating. The photocatalytic coating is suitable for applications to degrade benzene, toluene, ethylbenzene, and *o-m-p*-xylenes.^96^ Due to their outstanding thermal, mechanical, and durability properties, polymer matrix nanocomposites (PMCs) offer functional benefits for the construction industry. The addition of nanoparticles such as nanoclay, carbon nanotubes, graphene, nanosilica, or metal oxides into polymer matrices facilitates the creation of coatings with tailored properties, including self-cleaning, antimicrobial, and antifouling properties.^77,78^

#### 3.1.2 Metal-based nanocomposites

Metal matrix nanocomposites (MMNCs) are materials composed of a ductile metal or alloy matrix, which serves as a base for embedding nanoparticle reinforcements.^80^ The mechanical, chemical, and physical properties of these composites, consisting of a metal or alloy matrix filled with nanoparticles, are entirely different from those of the matrix material.^75^ Researchers have recently explored metal matrix nanocomposites for their exceptional properties, achieved through the dispersion of nanoparticles, which can be applied in various structural applications.^81^ These nanoparticles act as an obstruction to displacement movement, thereby improving mechanical features. Common methods for processing metal matrix nanocomposites include traditional techniques such as spray pyrolysis, liquid metal infiltration, vapor methods, rapid solidification, electrodeposition, and chemical processes such as colloidal and sol–gel methods.^82^

In another instance, Gu *et al.* developed a multilayer superhydrophobic coating using low-surface-energy silane, SiO_2_ particles, and epoxy resin. The coating exhibits excellent impermeability and durability, with a 92% improvement in chloride ion resistance compared with untreated concrete. In addition, enhancing the surface roughness or constructing micro-nanostructures on the surface can significantly augment the hydrophobic characteristics of the coating.^97^

#### 3.1.3 Carbon-based nanocomposites

Carbon nanocomposites (CNCs) are innovative materials that consist of carbon-based structures at the nanoscale, representing the intersection of carbon science and nanotechnology.^84^ CNCs are formulated by integrating carbonaceous materials with various nanostructures, which are critical in determining their properties and functionalities. Choosing and combining these components is vital for effectively customizing CNCs for specific applications.^85^ For instance, different carbon allotropes, such as graphene, carbon nanotubes (CNTs), and nanodiamonds, are integrated with other nanomaterials such as metal oxides, polymers, or nanoparticles to achieve the desired attributes.^86^ Nanocarbon materials, primarily allotropes such as graphene and its derivatives, have established a prominent role and are regarded as promising materials within the scientific community. Functionalizing these materials is crucial for enhancing their performance.^87^ In another instance, chemical functionalization of graphene as a nanomaterial involves modifying its surface, typically by introducing oxygen-containing groups such as hydroxyl, carboxyl, epoxy, and carbonyl *via* oxidation to produce graphene oxide (GO). This functionalization can be implemented either through covalent methods such as oxidation to graphene oxide or silane grafting or non-covalent approaches using polymers or superplasticizers, introducing surface functional groups that enhance its dispersion and interfacial bonding with cement hydration products.^98^

Graphene oxide (GO), as a promising 2D nano-material, is the product of chemical exfoliation of graphite that has homogenous and stable dispersion in aqueous media.^99^ Due to its good dispersibility in water and its high surface area of about 2600 m^2^ g^−1^, GO could be a potential alternative for the surface protection of concrete.^100^ In recent years, there have been some pioneering studies on the application of GO with building materials to improve their performances. Gong *et al.* reported that the introduction of 0.03% by cement weight of GO nano-sheets into cement paste increased its compressive strength and tensile strength by more than 40%.^101^ Similarly, Pan *et al.* described that the introduction of 0.05% by cement weight of GO into cement paste increased its compressive strength by 15–33% and flexural strength by 41–59%.^102^ Furthermore, Li *et al.* investigated the dispersion of GO in simulated pore solution and cement paste and observed severe GO aggregation in the presence of divalent calcium ions in both pore solution and cement paste.^103^

#### 3.1.4 Hybrid-material-based nanocomposites

Hybrid materials are highly valued for their exceptional mechanical strength, chemical stability, thermo-sensitivity, optical properties, corrosion resistance, and electrical and fire retardancy.^89^ This concept is further exemplified by hybrid organic-inorganic nanomaterials, which combine organic and inorganic components to achieve synergistic properties. Nanoparticles are particularly interesting because they promote the synergistic interactions between organic components of hybrid material coatings and inorganic compounds on heritage substrate materials, improving their protective capabilities and enhancing the coating performance. Nanosilica (n-SiO_2_), nano-titanium dioxide (n-TiO_2_), nano-zinc oxide (n-ZnO), carbon nanotubes (CNTs), graphene oxide, and nano-aluminium oxide (n-Al_2_O_3_) are the most popular materials, and hybrid materials containing combinations of these nanomaterials have been used for coating concrete structures.^26^ Common synthesis techniques include sol–gel processing, electrodeposition, and spray coating, all of which encourage uniform dispersion and effective adhesion. These materials are used in various industries, including aerospace, marine, automotive, healthcare, and electronics, offering advantages such as self-cleaning capabilities, antimicrobial protection, and UV resistance.^90^

Mahmoud *et al.* demonstrated a 74.6% reduction in water absorption rate by incorporating n-SiO_2_ and n-Al_2_O_3_ into a sol–gel system dispersed in a tetraethoxysilane (TEOS) polymer solution. The results reported by these authors were promising, achieving super-hydrophobicity with a maximum reported contact angle (CA) of 135°.^104^

## 4 Criteria for selecting suitable materials

Superhydrophobic surfaces have static contact angles greater than 150° and sliding angles less than 10°. It is well known that the wettability of a solid surface depends on two main factors: surface roughness and surface chemistry.^105^ The energy is determined by the chemical composition of the surface, which significantly influences its wettability. The primary method for creating superhydrophobic surfaces involves patterning roughness on a solid surface, followed by applying a thin layer of hydrophobic coating material.^106^ Hydrophobic chemistry typically relies on fluoropolymers or siloxanes with low surface energy.

Enhancing the durability of superhydrophobic surfaces is an important issue for self-cleaning and anti-corrosion properties. However, most of the superhydrophobic coatings obtained by the reported methodologies possess limited abrasion resistance.^107^ The evaluation of the change in static contact angle, contact angle hysteresis, and surface friction coefficient determines the abrasion resistance. Nanoparticles (NPs) usually regulate and control the nanoscale roughness of coated surfaces. SiO_2_ NPs, characterized by abrasion resistance, low-cost availability, and a variety of particle nano-sizes, are a common choice for fabricating transparent superhydrophobic surfaces.^108^

In another instance, Ramachandran *et al.* controlled entropic molecular interactions to design a novel icephobic concrete.^109^ This type of concrete showed a low ice adhesion strength and could repel incoming water droplets at −5 °C. Zhao *et al.* fabricated a superhydrophobic surface on concrete for anti-icing.^110^ She *et al.* developed a superhydrophobic concrete surface by spraying modified silica gel to simultaneously construct a hierarchical micro/nanostructure and lower the surface energy.^111^

Table 5 lists different nanocomposites used to develop superhydrophobic coatings, categorized by material type, synthesis methods, and outcomes. It includes various categories of nanocomposites, including thermoplastics, thermosets, elastomers, metals, carbon-based materials, and hybrid materials. This synthesis process produces coatings suitable for a range of industrial and functional uses.

**Table 5:** Overview of the synthesis processes of superhydrophobic coatings

Type of nanocomposite	Categories	Superhydrophobic coating	Synthesis process techniques	Result	References
Polypropylene/clay nanocomposites	Thermoplastic polymer nanocomposite	Surface roughness enhancement, low surface energy modification, spray coating or dip coating techniques	Clay modification, melt compounding, dispersion & exfoliation	Automotive coating, construction & architectural coating, aerospace coating	112
Polyethylene/silica nanocomposites	Thermoplastic polymer nanocomposite	Layer-by-layer (LBL) deposition, silane modification	Silica surface modification, Polyethylene and silica mixing, extrusion and cooling	Self-cleaning, anti-fogging, corrosion-proof	113
Epoxy/carbon nanotube nanocomposites	Thermoset polymer nanocomposite	CNT dispersion, coating deposition	Ultrasonication, high-shear mixing, three-roll milling	Electronics and electrical applications, energy storage and batteries	114
Vinyl ester/silica nanocomposites	Thermoset polymer nanocomposite	Surface modification of silica nanoparticles, dispersion of silica in vinyl ester resin	Sol–gel method, surface modification, dispersion of silica in vinyl ester resin, polymerization	Chemical and corrosion-resistant equipment, civil engineering and construction	115
Natural rubber/carbon black nanocomposites	Elastomer polymer nanocomposite	Surface roughness, low surface energy	Melt blending, *in situ* polymerization, sol–gel method, electrospinning method	UV protection & weather resistance, anti-static & conductive paints	116
TiO_2_ nanocomposites	Metal-based nanocomposite	Anti-corrosion & anti-icing coatings, durability improvement	Hydrothermal method, sol–gel method, precipitation method, electrochemical deposition, chemical vapor deposition	Paints and coatings, anti-fogging coatings	117
Al_2_O_3_ nanocomposites	Metal-based nanocomposite	Corrosion & chemical protection, electronic & sensor protection	Sol–gel method, Co-precipitation method, combustion synthesis	Wear-resistant coatings for electronic devices, self-cleaning & scratch-resistant coatings	118
Graphene-based nanocomposite	Carbon-based nanocomposite	High surface area, layer-by-layer assembly, spray coating	*In situ* polymerization, melt blending, exfoliation adsorption, chemical vapor deposition	Pollutant removal, coatings, filtration membranes	119
Carbon nanotube nanocomposites	Carbon-based nanocomposite	Low surface energy, surface roughness, layer-by-layer assembly, chemical vapor deposition	Carbon nanotube (CNT) synthesis methods, CNT functionalization, dispersion of CNTs, fabrication of CNT nanocomposites	Aerospace & automotive industry, biomedical & healthcare, structural & coating applications	119
Ni–TiO_2_ nanocomposites	Hybrid-material-based nanocomposites	Water-repellent & self-cleaning surfaces, antibacterial & antifouling coatings, anti-corrosion coatings, spray or dip-coating of nanoparticle suspensions	Sol–gel method, hydrothermal method, Co-precipitation method, electrochemical deposition	Photocatalysis & environmental remediation, hydrogen production & energy applications, catalysis & industrial applications	120
Cu-PDMS (polydimethylsiloxane) nanocomposites	Hybrid-material-based nanocomposites	Surface modification, fabrication methods, surface roughness	direct mixing method, *in situ* Cu nanoparticle formation in PDMS	Flexible & stretchable electronics, thermal management & heat dissipation, antimicrobial coatings & surfaces	121

Additionally, Table 6 comprehensively summarizes durability tests that are directly relevant to evaluating the long-term performance of superhydrophobic coatings for construction applications. These tests are highly relevant as they simulate real-world mechanical and environmental stresses that superhydrophobic surfaces might encounter. Sandpaper abrasion and friction wear (ball-on-plate) tests assess resistance to mechanical wear and surface damage caused by prolonged exposure to high-kinetic-energy water flows and impact damage from falling objects, which are critical challenges for building surfaces. The waterfall/jet test simulates prolonged rain exposure and high-impact water flow, verifying whether the coating can retain its water-repellent behavior under realistic environmental conditions where the kinetic energy of water can destroy the surface roughness and induce wetting. Adhesion-related durability, examined by tape peeling and pull-off strength tests, ensures strong bonding between the coating and substrate. Compared to many commercially available water-repellent coatings, the referenced results demonstrate a broader and more rigorous durability assessment, particularly in tribological and impact-related aspects.

**Table 6:** Factors demonstrating the wettability and durability of superhydrophobic coating materials

Factors	Significance	Overview of the case study	References
Wettability tests	• Greater than 150°, exhibiting excellent water-repellent properties and significantly reducing surface degradation	• The superhydrophobic coatings were created by spraying an SiO_2_-ethanol suspension onto a dip-coated epoxy resin film on a glass slide. The results indicated that the water contact angle (CA) of the coating reached 154.7°	110
Water contact angle (WCA) test	• It is considered when surfaces are not atomically smooth, contaminated, swelled, or reoriented		
Water sliding angle (WSA) test	• A sliding angle of less than 10° is a key characteristic of superhydrophobic surfaces	• Fabrication of porous polymer coatings by chemical vapor deposition resulted in a superhydrophobic coating that has a rigid elastic and micro-nanostructure; its CA was 157°, and sliding angle (SA) was 1°	122
	• Water droplets exhibit “rolling off” behavior with a low sliding angle, typically below 10°, due to a high water contact angle (greater than 150°) and low surface energy		
Contact angle hysteresis (CAH) test	• A contact angle hysteresis of less than 5° is ideal for making a surface superhydrophobic	• The theory is applied to predicting contact-angle hysteresis on rough surfaces from the hysteresis observed on smooth surfaces and therefore relevant to predicting roll-off angles for droplets on tilted surfaces	123
	• It accounts for the adhesiveness of a water droplet with the substrate, and it is the difference between the advancing angle (*θ*_A_) and the receding angle (*θ*_R_)		
Durability tests	• It can be used to monitor changes in water repellency over time	• Karsten tube tests revealed zero water penetration on hydrophobized samples	124
Karsten tube analysis test	• The liquid typically interacts very little with the surface due to the high degree of water-repellency, meaning it does not wet the surface but forms a droplet, resulting in minimal or no contact between the liquid and the coating		
Sandpaper abrasion test	Coated sandpaper with grit sizes of 240, 400, 800, 1000, and 1500 expresses super-hydrophobic properties. It exhibits a high water contact angle between 158° and 165° and a low sliding angle ranging from 10° to 2°	• The creation of superhydrophobic surfaces (SHS) increases the surface roughness. Sandpaper itself possesses an intrinsic surface roughness formed by abrasive particles, which are securely attached to its surface, reinforcing the robustness of its surface roughness	125
Water uptake test	• It is characterized by a combination of high surface roughness and low surface energy, which allows water to form droplets and roll off easily	• Hydrophobic SiO_2_ nanoparticles using tetraethyl orthosilicate, NH_4_OH, H_2_O and methyltriethoxysilane in ethanol, constructing a robust surface structure comprising substrate, bonding layer and superhydrophobic layer	62
	• Micro/nano hierarchical structures provide a valid method to strengthen the mechanical stability of superhydrophobic surfaces		
Corrosion resistance test	• Superhydrophobic surfaces create an air layer between the surface and water, which further minimizes contact with corrosive liquids	• The combination of superhydrophobicity and low conductivity is crucial for enhancing corrosion resistance. Additionally, the inclusion of SiO_2_ nanoparticles in a PTFE superhydrophobic coating enhances corrosion protection by filling the defects and pores within the coating	126
	• Minimizes contact between corrosive substances and the underlying material, thus slowing down or preventing corrosion		
Water impact resistance test	• Water-repellent capacity depends on the micro- or nano-structured surface of the coating, which is essential for superhydrophobicity	• Altering a magnesium oxychloride cement composite with a superhydrophobic coating greatly enhanced its water resistance	127
	• Some superhydrophobic coatings, especially fluoropolymer-based ones, can withstand moderate water impact		

## 5 Fabrication techniques for superhydrophobic coatings on concrete

Concrete is naturally hydrophilic, allowing water and corrosive ions to easily penetrate its pores, which diminishes the durability of structures.^128^ To combat this, superhydrophobic modification is commonly employed to reduce/limit water infiltration and enhance durability.^129,130^ Most superhydrophobic coatings are developed by creating rough surfaces through multistep processes.^131^ Recent studies categorize concrete surface treatment methods into three primary types: surface coatings, pore-blocking treatments, and hydrophobic impregnation.^132^ Finding suitable materials, simple fabrication techniques, and efficient, durable, eco-friendly superhydrophobic coatings for practical use remains challenging.^133^ Based on super-hydrophobicity principles, various strategies have been devised to create concrete surfaces with suitable topological structures and surface chemical compositions to achieve water repellency.

### 5.1 Deposition methods for coating materials

**Fig. 8:**
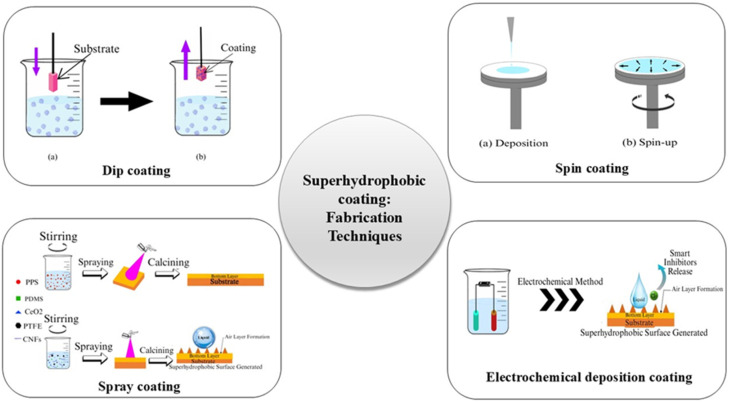
Different types of fabrication techniques of superhydrophobic coatings.

The development of superhydrophobic systems has attracted increasing attention because of their numerous practical applications.^134^ These fabrication techniques are generally categorized into four main methods: (1) dip coating by immersing concrete samples into hydrophobic organic solutions; (2) spin coating with a polymer or polymer emulsion mixed with cement applied on the surface; (3) spray coating hydrophobic suspensions enriched with micro/nano particles on the surface; and (4) electrochemical deposition by replicating the delicate micro- and nano-scale surface structures^128^ shown in Fig. 8. These approaches aim to reduce the surface energy and create hierarchical roughness, which enhances water the contact angles and minimizes water absorption. Table 7 briefly compares different fabrication techniques for superhydrophobic coatings with a clear focus on their potential suitability for construction/building applications. Implementing these coatings in the real world is a challenging task. Most of these techniques can only be applied to lab samples, with very few being practical for large-scale infrastructure.^135^

**Table 7:** Comparative analysis of the fabrication techniques of advanced concrete coatings

Fabrication technique	Process of fabrication	Materials used	Advantages	Suitability for construction application	References
Dip coating technique	Substrate is immersed in a hydrophobic solution and withdrawn at a controlled speed	Sol–gel systems, polymeric coatings	Low processing temperature, good adhesion, uniform coating on all sides, minimal waste, durable, compact	Suitable only for small components (meshes, fences, small metal parts)	68 and 136
Spin coating technique	Solution is dispensed on a flat substrate, which is spun at high speed (1000–8000 rpm) to spread into a thin uniform film	Hydrophobic electrospun fibers using thermoplastic elastomers	Excellent uniformity and thin films; good control of thickness *via* speed	Not feasible for building infrastructure, lab-scale only	115, 137 and 138
Spray Coating technique	Hydrophobic suspension is sprayed onto the surface using a spray gun	Polymer solutions, silanes, nanocomposites	Simple, fast, low-cost, automatable, repairable	Highly practical & scalable for large areas *e.g.*, concrete, steel, bridges	111 and 139
Electrochemical deposition technique	Uses anodic oxidation, galvanic deposition, or anodization to grow micro/nano structures	Metals, alloys, conductive polymers, and ceramics	Precise control of morphology	Promising for corrosion protection, but scalability is limited for large concrete elements	140 and 141
Chemical vapor deposition (CVD) technique	Gas-phase chemical reactions forming thin films	Silanes, fluorosilanes	Highly uniform, durable coatings	Not practical for large concrete surfaces	142
Layer-by-layer (LbL) technique	Alternate adsorption of oppositely charged species	Polymers, nanoparticles	Precise thickness control	Laboratory-based only	143

#### 5.1.1 Dip coating technique

Dip coating is a valuable technique for fabricating superhydrophobic coatings. This process involves continuously dipping the substrate into a solution containing the desired deposition material at a constant speed.^144^ When the substrate is immersed in the solution for a specific duration and lifted out, a film is deposited it as it is removed.^68^ The withdrawal or pulling speed determines the film thickness; typically, a higher speed results in a thinner film. Excess solution is removed from the surface during withdrawal.^145^ The solvent in the solution evaporates, leaving a coating on the surface. This technique has no self-repairing ability. It involves immersing the substrate into a coating solution and then slowly withdrawing it, allowing a thin, uniform film to form as the solvent evaporates. It is widely used for superhydrophobic coatings because it is simple, low-cost, scalable, and effective for complex or porous surfaces, including concrete or fabrics.^146^

This method offers numerous benefits, including the ability to coat both the top and bottom parts of the substrate simultaneously, minimal material waste, compatibility with various materials, high production output, uniform coatings, and coatings that are highly durable, compact, stable, and easily repairable.^68^

#### 5.1.2 Spin coating technique

The spin coating technique generates a thin layer on relatively flat substrates. A solution of the material to be applied is placed onto the substrate, which is then rotated at high speeds ranging from 1000 to 8000 rpm, resulting in a uniform coating.^147^ The thickness of the deposited film is determined by the angular velocity, solution viscosity, and spinning duration. The thickness of the film can be modified by adjusting the spin speed or using various photoresists. This technique is particularly effective on a laboratory scale.

#### 5.1.3 Spray coating technique

In this method, a solution of the coating materials is applied to the substrate using a spray gun. The method generally begins with thorough surface preparation of the concrete, involving high-pressure washing to remove dust and oils, followed by drying and optional light abrasion for better adhesion. Next, a coating suspension is prepared by dispersing nanoparticles in a solvent such as ethanol or isopropanol, incorporating a low-surface-energy modifier such as superhydrophobic agent and an adhesive such as epoxy resin. Application occurs *via* spray coating using a low-pressure spray gun or airbrush.^69^ This approach offers several benefits, including simplicity, wide availability, potential for automation, speed, cost-effectiveness, ease of repair, and non-toxicity.^139^ Additionally, this technique can be utilized on various substrates such as plastics, metals, and fabrics.^148^

#### 5.1.4 Electrochemical deposition technique

The electrochemical deposition method stands out as one of the most promising approaches due to its ability to easily control the kinetics of surface feature growth and create diverse morphologies over large surfaces.^140^ Electrodeposition is an electrochemical phenomenon that underpins an emerging class of treatments with unique potential to rehabilitate concrete structures. Practical implementations include laboratory-validated field trials and actual repairs of cracked or corroded reinforced concrete, demonstrating effective crack closure, improved corrosion resistance, and minimal pH disruption when using variable or reversed polarity currents for more uniform deposition.^149^ However, it is not commonly used for superhydrophobic coatings on concrete surfaces. Superhydrophobicity is typically achieved *via* spray, spin, or sol–gel methods with nanoparticles. These electrochemical processes are facile, rapid, and reproducible. Various electrochemical deposition techniques including anodic oxidation, galvanic deposition, electroplymerization, and electrochemical anodization are commonly employed for material production.^150^

### 5.2 Scalable fabrication approaches

This section will explore superhydrophobic coatings made from different green materials, highlighting their chemical, physical, and environmental considerations. Table 8 shows that nanoparticles have diverse applications, ranging from self-cleaning surfaces and corrosion protection to antibacterial activity, UV shielding, and eco-friendly coatings. Green materials include sepiolite, halloysite, PDMS, ZnO, cinnamic acid, myristic acid, *etc.*, modified by octadecylamine, corn husk, and rice husk, yielding nanoparticles such as silicate clay, silica, alumina, gold and silver, metal oxide, and solid lipid nanoparticles, among others.^151^ These green materials are agricultural byproducts that can be used as superhydrophobic coating materials for concrete as they are environmentally friendly, cost-effective, and durable. Sepiolite and halloysite are fibrous and tubular clay minerals with abundant silanol (Si–OH) groups and a porous structure, which provide a high specific surface area and active sites for modification, enabling the creation of rough surfaces essential for superhydrophobicity.^152^ Their capacity for cation exchange and surface functionalization allows for the stable incorporation of hydrophobic agents, such as octadecylamine, which lowers the surface energy by introducing long hydrocarbon chains, enhancing the water repellence. PDMS (polydimethylsiloxane) serves as a flexible, durable binder that supports the micro-nano roughness needed for superhydrophobicity while offering chemical and mechanical stability.^142^ ZnO, cinnamic acid, and myristic acid contribute to surface roughness and hydrophobicity through their inherent chemical properties. Additionally, agricultural waste such as corn husk and rice husk provides eco-friendly, renewable fillers that impart hierarchical surface textures and reduce environmental impact.^143^

**Table 8:** Applications of green material-based superhydrophobic coatings in surface modifications

Green materials	Nanocomposite incorporating superhydrophobic coating agents	Nanoparticle type	Application	Overall result	Cost & scalability	References
Sepiolite	Fatty acids, organosilanes	Silicate clay mineral	Self-cleaning and corrosion protection	Enhances their performance by contributing to robustness, mechanical stability	Low cost; scalable	7
Halloysite	Organosilanes, polymers	Silica and alumina	Create rough surfaces, achieve extreme water repellency	Contributes to the overall hydrophobicity of the coating, leading to higher water contact angles	Low-moderate cost; scalable	151
PDMS	SiO_2_, TiO_2_	Silver and metal oxides	To enhance adhesion to the substrate and impart hydrophobicity	Exhibits good durability and stability, resisting abrasion, UV radiation, and harsh chemical environments	Moderate cost; scalable	143
ZnO	Stearic acids, silanes	Metal oxide nanomaterial	Decrease the surface energy and increase the surface roughness by reacting with the mixed solution	High water contact angles (WCA), self-cleaning capabilities, and potential antibacterial and UV-blocking effects	Low cost; highly scalable	153
Cinnamic acid	Fluorinated polymers, silanes	Zinc oxide (ZnO) nanoparticles	Antibacterial activity and UV shielding, making surfaces water-repellent	Excellent robustness and water-repellence properties, materials with low surface energy	Moderate cost; scalable	152
Myristic acid	SiO_2_, TiO_2_, or Al_2_O_3_	Solid lipid nanoparticles (SLNs)	To modify surfaces and create water-repellent properties	Becomes highly water-repellent, with water droplets beading up and rolling off easily	Low cost; scalable	142
Bagasse	Dimethyldiethoxysilane (DMDEOS), fatty acids, fluorinated compounds	Silica nanoparticles	Good adhesion and good protective properties that allow it to withstand the diffusion of water vapor	Good anti-fogging, anti-freeze, and anti-bacterial properties	Low cost; highly scalable	154
Egg waste and stearic acid (STA)	SiO_2_, TiO_2_, ZnO, MgO	Calcium oxide (CaO) and solid lipid nanoparticles	Environmentally friendly material for fabricating a hydrophobic barrier	Superhydrophobic properties, 4° sliding angle, mechanical resistance towards damage and UV irradiation	Very low cost; scalable	155
Chitosan modified by octadecylamine	Octadecylamine (ODA), fatty acids, fluorinated compounds	Fe_3_O_4_	To create durable and sustainable superhydrophobic coatings, particularly for textiles	Superhydrophobic properties superoleophilic properties	Moderate cost; scalable	156
Corn husks	Perfluorodecyltriethoxysilane (PTES), PVA (polyvinyl alcohol)	Silica, cellulose nanocrystals, and even metal nanoparticles such as palladium, silver, and copper	Preventing corrosion and rust	De-icing properties, photothermal properties	Low-moderate cost; scalable	23
Rice husk ash	Fluoroalkyl silane (FAS), 1*H*,1*H*,2*H*,2*H*-perfluorodecyltriethoxysilane	Silica nanoparticles	To create a rough surface, enhancing water repellency and other properties	High water repellency (water contact angles exceeding 150°) and reducing water uptake in materials such as concrete	Low cost; highly scalable	157
Wheat straw	Stearic acid, fatty acids, or silane coupling agents	Nanocellulose, nanolignin, nanosilica, silver nanoparticles	Self-cleaning surfaces	High water repellent properties	Low cost; scalable	113

## 6 Tribological performance and durability of nanocomposite-derived superhydrophobic coatings under mechanical stress on concrete materials

**Fig. 9:**
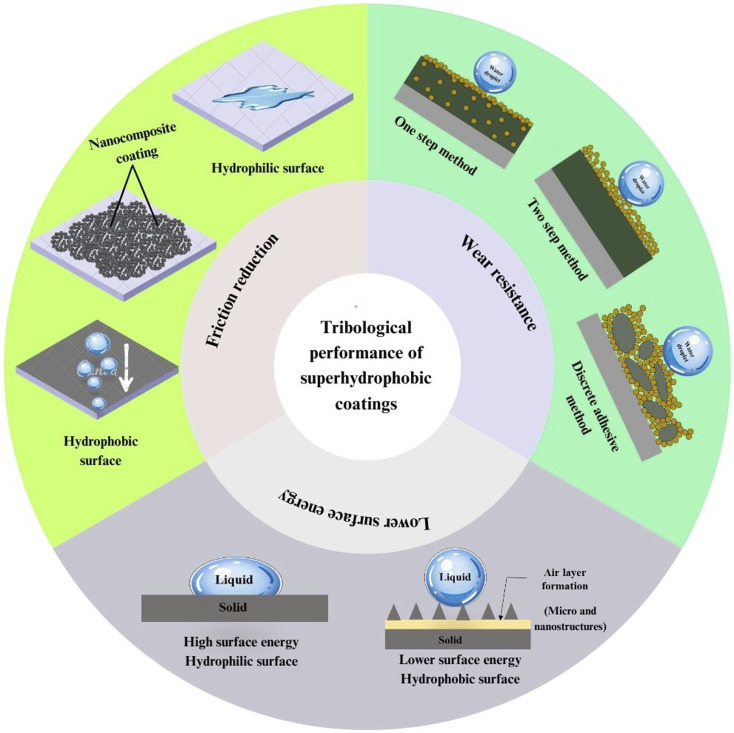
Schematic of the tribological performance of superhydrophobic coatings.

Due to its extremely non-wetting properties, a superhydrophobic surface has a wide range of potential applications, including wear resistance, drag reduction, anti-corrosion, anti-icing, and self-cleaning.^158,159^ The long-term effectiveness of superhydrophobic coatings on concrete structures is critically dependent on their tribological performance, including friction, wear resistance, and surface contact mechanics, as shown in Fig. 9.^160,161^ It illustrates the interplay among surface chemistry, nanocomposite coating structure, and wettability. Surface roughness and low surface free energy are the two key factors in creating a superhydrophobic surface, which are also the main reasons for its excellent tribological performance.^162^

Table 9 comprehensively summarizes the durability tests that are directly relevant to evaluate the water repellent properties of superhydrophobic coatings for construction applications. These tests are particularly relevant as they simulate real-world mechanical and environmental stresses on superhydrophobic surfaces. The sandpaper abrasion and ball-on plate friction wear tests specifically asses resistance to mechanical wear and surface damage caused by prolonged exposure, high-kinetic energy water jets, impacts from falling objects, and other wear factors critical to building applications. The waterfall/jet test simulates prolonged rain exposure and high-impact water flow, verifying whether the coating can retain its water-repellent behavior under realistic environmental conditions where kinetic energy of water can destroy surface roughness and induce wetting. Adhesion-related durability, examined by tape peeling and pull-off strength tests, ensures strong bonding between the coating and substrate. Compared to many commercially available water-repellent coatings, the referenced results demonstrate a broader and more rigorous durability assessment, particularly in tribological and impact-related aspects.

**Table 9:** Durability tests of superhydrophobic coatings to evaluate their water repellent properties

Durability tests	Properties	References
Sandpaper abrasion test	It uses a controlled abrasive to measure how the surface of a material withstands wear	163
Friction wear test (ball-on-plate tribometer)	This method uses a tribometer to slide a ball or stylus across the surface under a controlled load and motion	164
Waterfall/jet test	It detects surfaces under long-term water exposure and how different kinetic energies of water impact the surface influence wetting properties	165
Falling weight impact test	A weight is dropped onto the surface to assess its resistance to cracking or delamination	166
Tape peeling test	An adhesive tape is applied and removed, and the surface is inspected for coating removal	167
Pull off strength test	A quantitative method where a dolly is adhered to the coating and pulled off with a controlled force	163

### 6.1 Tribological mechanisms in superhydrophobic nanocomposites

Superhydrophobic nanocomposites, particularly formed through the self-assembly of nanostructures such as inorganic micro- and nano-particles or carbon nanotubes, exhibit enhanced tribological properties that contribute to their practical applicability.^168^ The development and further enhancement of high-performance, wear-resistant materials will create significant economic and social benefits.^169^ Enhancing the tribological properties of materials to reduce wear and friction during the work process can boost the efficiency and prolong the lifespan of machinery, thereby lowering carbon dioxide emissions and reducing resource and energy waste in production and manufacturing processes.^170^ Mechanical wear directly affects superhydrophobicity by progressively damaging the hierarchical micro–nano surface structures and/or removing the low-surface-energy chemical groups that are essential for maintaining the Cassie–Baxter wetting state. During abrasion or sliding, the uppermost nano-features are typically worn first, leading to a gradual reduction in surface roughness and trapped air fraction. As a result, the water contact angle (WCA) decreases, while the sliding angle (SA) and contact angle hysteresis (CAH) increase.^171^

According to Mohammed *et al.*, zirconia (ZrO_2_) nanocoatings exhibit excellent tribological characteristics and are also attractive for their electrical and optical properties.^172^ In another instance, Zhang *et al.* evaluated the deposition of nano-ZrO_2_ film coatings on 304 stainless steel substrates. The nanofilms showed reduced friction coefficients when tested against an SiC grinding ball in 5% NaCl solution, distilled water, and dry conditions. Under dry conditions, adhesive and oxidation wear were the main wear mechanisms, whereas in 5% NaCl solution, corrosive wear was dominant. The coatings exhibited significantly reduced friction coefficients (≈0.25–0.35) compared to the uncoated substrates.^173^

Gu *et al.* examined that alumina (Al_2_O_3_), an oxide ceramic, has been extensively utilized as a coating material owing to its superior wear resistance. The wear behavior of nanostructured alumina coatings on SS304 stainless steel substrates, created *via* atmospheric plasma spraying, was assessed and compared to that of micro-alumina coatings. The nano-alumina coatings exhibited better wear resistance than the micro-coatings under applied normal loads of 30 N to 80 N. Graphene- and CNT-reinforced epoxy nanocomposites, frequently proposed for concrete protection, were evaluated using steel or alumina balls under normal loads of 5–15 N, sliding speeds of 0.05–0.1 m s^−1^, and sliding distances of 100–500 m.^174^

The use of carbon nanocomposites such as graphene and carbon nanotubes (CNTs) in epoxy composites has revolutionized the field of tribology by significantly enhancing the mechanical and tribological properties of these materials.^175^ Incorporating graphene into the epoxy matrix improves its mechanical strength and toughness; the high modulus of elasticity of graphene aids in better load distribution, preventing localized stress concentrations that can lead to wear and failure.^176^ Studies indicate that graphene-reinforced epoxy exhibits significantly lower wear rates compared to unmodified epoxy. Furthermore, graphene's exceptional tensile strength enhances the load-bearing capacity of epoxy composites, that helps to maintain structural integrity under mechanical stress and amke these materials ideal for applications in concrete industries.^177^ This mechanism provides superior tribological performance on concrete surfaces through synergistic combination repellency-driven contact minimization, robust interfacial adhesion, and partial integration into the substrate. However, long-term durablity remains limited by substrate erosion in highly porous or low-strength concretes under severe mechanical loading.^169,178^

### 6.2 Mechanical durability and retention of super-hydrophobicity post-stress

The long-term durability of self-assembled nanostructures against mechanical wear, friction, and surface contacts is essential for their practical application in superhydrophobic surfaces.^111^ Surfaces with convex, resilient micro and nano features, such as those created through hydrothermal treatments or biomimetic designs, generally preserve high contact angles (above 150°) and low sliding angles, even after hundreds of abrasion cycles.^161^ Coatings anchored to hydrophobic nanoparticles and elastomeric matrices can retain or regenerate surface roughness during mechanical deformation, thereby enhancing the abrasion resistance.^179^ Dominant failure mechanisms after mechanical stress, such as abrasion, friction wear, sandpaper tests, tape peeling, or impact, in durability assessments in nanocomposite-derived superhydrophobic coatings typically include the loss of surface roughness.^180^

According to Gemici *et al.*, the extremely thin (100 nm) and highly porous nature of hydrothermally fabricated nanoparticle-containing films most likely enhanced their scratch resistance compared with films prepared *via* a high-temperature calcination process. For superhydrophobic silica nanoparticle coatings, their initial WCA was 165°–170° with SA of ∼2°. After scratch testing (equivalent to 50–100 abrasion cycles at 100–200 g load), the WCA decreased to 150°–155° and the SA increased to 5°–8°; however, the films retained non-wetting properties due to their porous, resilient structure.^181^ Jung *et al.* evaluated the mechanical durability of a composite superhydrophobic carbon nanotube coating using epoxy as the matrix.^182^ The initial WCA was ∼160° with the SA of <5°. After cyclic wear tests using atomic force microscopy (100 nN load) and ball-on-flat tribometry, the coating retained super-hydrophobicity (WCA > 150°, SA < 10°), with no significant delamination or loss of adhesion to the substrate. Results indicated that the coating retained its adhesion to the substrate and maintained superhydrophobic properties after undergoing cyclic wear tests, which ranged from 100 nN pressure in atomic force microscopy to 10 mN using a ball-on-flat tribometer.^18^

## 7 Techno-economic aspects and process analysis of coating materials

**Fig. 10:**
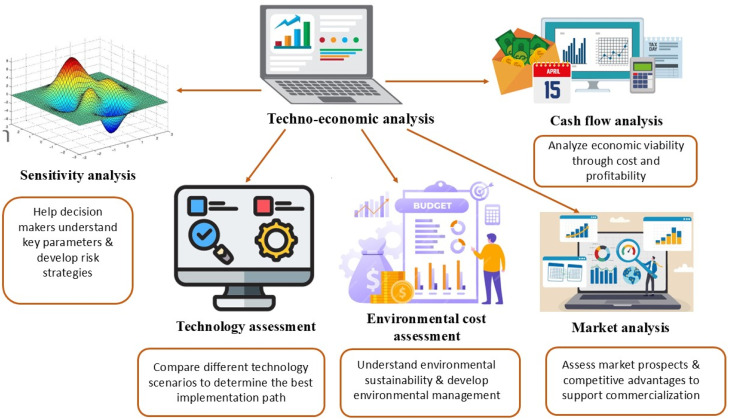
Techno-economic analysis of coating materials.

Techno-economic analysis (TEA) is essential for developing superhydrophobic coating materials, as it assesses their feasibility, practicality, and cost-effectiveness. It analyzes the cost-performance balance, including application and material expenses, alongside performance advantages such as durability, water resistance, and lower maintenance needs.^183^ Superhydrophobic nanocomposites frequently utilize advanced materials and manufacturing processes, which can be costly.^110^ TEA aids in examining the costs related to raw materials, production, labor, and equipment, providing valuable information on the economic viability of transitioning from laboratory-scale to industrial-scale production (Fig. 10).^184^

Zhou *et al.* created hydrophobic anti-icing concrete by incorporating a water-based stone protector and metakaolin. This altered concrete demonstrated outstanding durability, with its de-icing (the process of removing snow, ice or frost from a surface) pressure being approximately 1/16 and 1/22 that of standard concrete and rock, respectively. Additionally, the study confirmed that this material is feasible for large-scale applications in terms of both practicality and cost-effectiveness, making it advantageous for removing ice from tunnel linings and slope rock masses during winter.^82^

## 8 Superhydrophobic coatings for the protection of concrete structures: successful case studies

Superhydrophobic coatings fabricated with suitable roughness patterns and low surface energy materials exhibit various functional effects in self-cleaning, anti-fogging, anti-fouling, drag reduction, anti-corrosion, *etc.*^185^

**Fig. 11:**
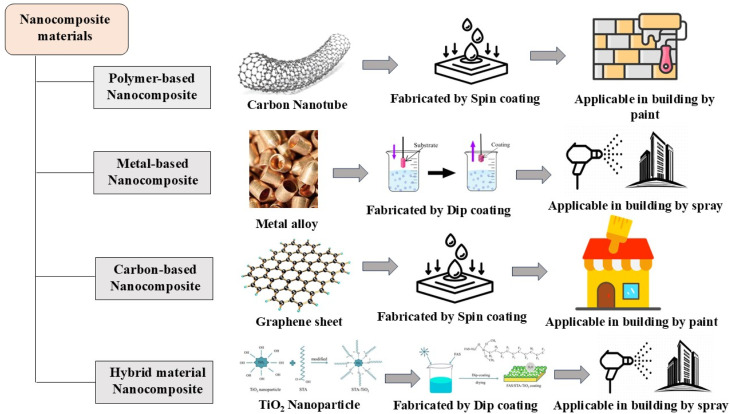
Application of various nanocomposite materials in concrete structure coatings.

Maintaining the exterior surfaces of concrete structures is crucial for preserving their aesthetic appeal and ensuring they remain durable in practical applications. Especially when compared to modern structures, the conservation and restoration of historical buildings made of brick, wood, and natural stones such as marble is essential.^186^Fig. 11 illustrates how the flexibility of coating formulas based on TiO_2_ nanoparticles and other materials provides different substrates with super-hydrophobicity and imparts additional practical and functional properties, such as excellent UV resistance.^187^ A blend of silica nanoparticles with polyurethane (PU) or epoxy resin can significantly enhance the mechanical durability of superhydrophobic coatings due to their superior adhesion strength to substrates.^188^

In another study, Xu *et al.* prepared a hydrophobic coating for cement mortar by adding stearic acid-modified superhydrophobic zeolite powder to reduce the surface energy, which significantly reduced the capillary water absorption of the mortar and improved its anti-erosion properties.^189^ Song *et al.* developed a fluorine-free superhydrophobic cement concrete surface coating that exhibited high surface mechanical strength and remained superhydrophobic after multiple scrapings or sandpaper abrasion for 20 m.^190^

Aslanidou *et al.* sprayed a mixture of SiO_2_ nanoparticles and an emulsion containing silane, siloxane, and organic polymer onto white marble and grey sandstone. This treatment was designed to impart both superoleophobicity and super-hydrophobicity properties, helping to keep the surfaces clean and visually appealing.^191^

## 9 Technical challenges in commercial production of superhydrophobic coating materials

The scalability and uniformity of superhydrophobic coatings for building applications face significant challenges primarily due to mechanical durability and low surface energy.^192^ The application process itself, whether by spraying, dipping, or brushing, can lead to uneven coverage.^129^ However, maintaining the nanoscale structure required for superhydrophobicity uniformly over a large surface is often cost-prohibitive. Environmental factors such as heavy rainfall, UV exposure, temperature fluctuations, and mechanical abrasion further complicate efforts to ensure long-term uniformity and scalability.^131^

### 9.1 Challenges in scalability

Many fabrication methods involve complex, multi-step processes or expensive materials, making large-scale production economically unviable. Consequently, transitioning from laboratory-scale synthesis to industrial-scale manufacturing often increases costs.^157^ The required micro/nanostructure and chemical composition for super-hydrophobicity can be difficult to replicate consistently over large areas.^156^ The fabrication of superhydrophobic surfaces by techniques such as electrospinning, chemical vapour deposition, and plasma treatments can be effective, but the significant scalability problems are primarily due to the difficulty in maintaining reliable surface roughness and chemical composition over huge areas. Variations in coating thickness, structure, or composition can result in incompatible superhydrophobic performances,^193^ which is compounded by the widespread use of fluorinated compounds in traditional coatings that pose significant environmental hazards.^194^ The selection of coatings is largely based on short-term laboratory testing results, with a limited understanding of their long-term performance in real-world environments. For example, while penetrating hydrophobic coatings demonstrate excellent chloride ion resistance in laboratory experiments, their effectiveness on concrete applications may be significantly reduced due to insufficient application thicknesses or improper substrate preparation.^195^ Therefore, the development of sustainable and eco-friendly alternatives is essential to support the large-scale implementation of superhydrophobic surfaces.

### 9.2 Challenges in uniformity

Concrete structure protection materials require uniformity in superhydrophobic coatings, mainly due to the minimal balance required between achieving the appropriate micro- and nano-scale surface structure and ensuring mechanical integrity.^196^ Environmental factors such as temperature and humidity during application can impact the coating performance. Mechanical stability issues complicate this problem, where adhesives or elastic materials can create weak points vulnerable to abrasion, UV degradation, or thermal expansion mismatch in outdoor environments.^134^

## 10 Future perspectives

Next-generation superhydrophobic coatings are being developed to overcome traditional limitations, including poor mechanical strength, accelerated aging, and environmental degradation.^124^ Applying superhydrophobic treatments to coating materials significantly enhances their waterproofing and impermeability, as well as their resistance to chloride ion penetration, freeze–thaw cycles, and other environmental factors. Future developments are likely to focus on environmentally benign formulations using bio-based polymers, aligning with green building standards and sustainability goals.^15^ Furthermore, innovative coatings with embedded nano-sensors could enable real-time monitoring of structural health, allowing for predictive maintenance and prolonging service life.^25^

### 10.1 Durability enhancements

A primary emphasis is on extending the lifespan and strength of these coatings. This includes creating materials and methods capable of enduring mechanical abrasion, chemical interactions, and environmental pressures.^197^

### 10.2 Eco-friendly coatings

There is a growing emphasis on replacing traditional fluorinated chemicals with sustainable alternatives, recognized for their environmental damage. Materials and coatings inspired by nature and sourced from biomass are quickly gaining popularity.^110^

### 10.3 Scalability and cost reduction

Many advanced manufacturing techniques, including chemical vapor deposition (CVD) or specialized etching methods, can produce high-performance superhydrophobic coatings.^70^ However, these techniques are costly and challenging to scale, restricting the practicality and economic viability of superhydrophobic coatings in commercial settings. Superhydrophobic coatings have a transformative future, with applications that enhance durability, eco-friendliness, and multi-functionality to make them widely accessible and sustainable.

### 10.4 Customization and application-specific coatings

Tailored coatings designed for specific building materials and environmental conditions will enhance the performance and foster adoption in diverse architectural contexts.^133^

## 11 Conclusion

Next-generation nanocomposite-based superhydrophobic coatings represent a transformative approach to sustainable heritage and concrete structure protection. By combining advanced nanostructured materials with durable, multifunctional properties, these coatings offer exceptional water repellency, self-cleaning capabilities, and resistance to environmental degradation. Their ability to prevent water infiltration, inhibit microbial growth, and reduce surface erosion makes them particularly valuable for preserving cultural heritage while ensuring long-term structural integrity. Despite the challenges associated with large-scale applications, uniformity, and long-term durability, ongoing research is paving the way for the wider application of scalable, eco-friendly, and adaptive coating technologies. Conventional superhydrophobic coatings for concrete often rely on fluorinated compounds (*e.g.*, fluoroalkylsilanes) combined with simple hydrophobic intrusions or basic nanoparticle embeddings. These coatings rely mainly on micro-scale roughness and hydrophobic surfaces to achieve high water contact angles. However, the use of harmful fluorinated materials and their poor mechanochemical stability limit their practical application. In contrast, next-generation nanocomposite-based superhydrophobic coatings emphasize sustainable, eco-friendly, and high-performance innovations, including non-fluorinated modifiers (*e.g.*, stearic acid, polydimethylsiloxane/PDMS or alkyl silanes), bio-based materials (*e.g.*, lignin and cellulose derivatives), and hierarchical micro/nano structures *via* advanced spray or sol–gel methods. It is important to remember that the traditional paint and coating industry has also developed many high-performance coatings that are as durable and effective as solvent-based paints or oil-based paints. Major hydrophobic acrylics and polyurethanes, silicones, waxes, and oils disperse easily in water and can easily coat almost any surface. Most studies have employed different techniques to assess the mechanical durability of superhydrophobic coatings, including sandpaper abrasion, adhesive tape peeling, and finger abrasion, among others. Nevertheless, there is a dearth of standardized methods for evaluating the durability of these coatings. In future works, more attention should be paid to improving durability and developing better durability test methods simulating the real conditions under which superhydrophobic coatings are subjected.

## Conflicts of interest

There are no conflicts to declare.

## Data Availability

No primary research results, software or code have been included and no new data were generated or analysed as part of this review.
